# Deep venous thrombosis in the antenatal period in a large cohort of pregnancies from western India

**DOI:** 10.1186/1477-9560-5-9

**Published:** 2007-07-04

**Authors:** Sonal Vora, Kanjaksha Ghosh, Shrimati Shetty, Vinita Salvi, Purnima Satoskar

**Affiliations:** 1 Department of Haemostasis, Institute of Immunohaematology (ICMR), KEM Hospital, Parel, Mumbai 400 012, India; 2 Department of Obstetrics & Gynaecology, KEM Hospital, Parel, Mumbai 400012, India; 3 Dept. of Obstetrics & Gynaecology, Nowrosjee Wadia Maternity Hospital, Parel, Mumbai 400 012, India

## Abstract

**Background:**

Deep venous thrombosis (DVT) is an important complication in the peripartal and postpartal period.

**Methods:**

We followed up prospectively the prevalence of DVT in 34720 prenatal mothers between June 2002 and July 2006 attending the antenatal clinics of two major hospitals in Mumbai, India. Thirty two women (0.1%) presented for the first time with symptomatic DVT i.e. 17 in the first trimester, 6 in the second and 9 in the third trimester of pregnancy. Nine had history of fetal loss while in the remaining twenty three there was no history of fetal loss.

**Results:**

The evaluation of both acquired and heritable thrombophilia showed a conglomeration of thrombophilia in this group when compared to 100 normal pregnant women who have given birth to at least one healthy baby with no history of fetal death, DVT or other obstetrical complications. The relative risks for all the antiphospholipid antibodies (APA) studied i.e lupus anticoagulant (LA), IgG/IgM antibodies for cardiolipin (ACA), β2 glycoprotein 1 (β2 GP 1) and annexin V were significantly higher in women with pregnancy associated DVT (RR 7.4 95% CI 4.3–11.3 P < 0.05). Among the genetic thrombophilia markers studied, Protein S (PS) deficiency was the strongest risk factor (RR 5.00 95% CI 3.02–5.00 P < 0.05) followed by factor V Leiden (FVL) mutation (RR 4.57 95% CI 2.23–4.57 P < 0.05) and PAI 4G/4G homozygosity (RR 3.24 95% CI 1.85–5.12 P < 0.05). Protein C (PC) and endothelial protein C receptor (EPCR) 23 bp insertion polymorphism was also increased in the patient group as compared to controls but the difference was not statistically significant. The MTHFR C677T, fibrinogen gene β448 Arg/Lys polymorphisms were not significantly different from the normal controls, while antithrombin III (AT III) deficiency and PT G20210A polymorphism were absent in both controls and patients. Two or more risk factors were present in 22 out of 32 cases (68.75%).

**Conclusion:**

We conclude that the prevalence of DVT in India is more or less similar to other reports published and both acquired and heritable thrombophilia show strong association with DVT associated with pregnancy.

## Background

The incidence of venous thrombosis in pregnant women is approximately 1 in 1000 – 20000 pregnancies [1]. It is an uncommon but the leading cause of mortality and morbidity in antenatal women.

During normal pregnancy, a series of changes take place in the coagulant and anti-coagulant pathways of blood coagulation as well as in the fibrinolytic pathway [2,3]. The plasma concentration of several proteins and their activities change and there is a hemostatic imbalance towards a prothrombotic state. Placenta and myometrium also contribute to a high concentration of a specific fibrinolytic inhibitor i.e PAI II during normal pregnancy [4]. In addition, the hormonal changes which occur during pregnancy tilt the thrombohaemorrhagic balance towards thrombosis to prevent severe postpartal blood loss. In spite of all these changes postpartal venous thrombosis is several times more common than antenatal venous thrombosis [5,6].

The presence of thrombophilia is an added risk factor for venous thromboembolism (VTE) during pregnancy. Except one report on postpartal cerebral venous thrombosis [7] there are no data on the prevalence of DVT during pregnancy in India. Neither there are any data on the risk assessment in women presenting with DVT for the first time during pregnancy. The present study was thus aimed at finding the prevalence and the risk associated with VTE in antenatal mothers attending two of the largest hospitals in Mumbai during the last four years.

## Methods

### Patients

The study was carried out between June 2002 and July 2006 on 34720 pregnant women attending the outpatient Departments of Obstetrics & Gynaecology, KEM Hospital and Nowrosjee Wadia Maternity Hospital, Mumbai, India. Clinical examination for symptomatic DVT was carried out by two Gynaecologists independently. DVT was documented by venography, Doppler ultrasound angiography, or MRI. None of the patients with confirmed DVT were neither receiving any anticoagulant therapy at the time of study. Only two patients had positive family history of DVT among first degree relatives.

### Controls

One hundred unrelated age matched pregnant women with the same ethnic background as that of the pregnant women with DVT were selected as controls for this study. Majority of them were Marathas while very few were Gujaratis, South Indians, Muslims and others, in both the groups. The control group included more or less equal numbers in the first second and third trimesters. None of the controls had a previous history of miscarriage or pregnancy complications and had at least one healthy baby at the time of this study. Women with endocrinological dysfunction, chromosomal abnormalities, autoimmune disease, liver dysfunction were excluded from both the patient and control group.

None of the patients who had DVT and the controls had any other confounding factors like smoking or history of taking oral contraceptives.

The study was approved by the Institute Ethics Committee.

### Methods

Blood was collected in 3.13% trisodium citrate (9:1 blood to anticoagulant). The platelet poor plasma was separated by centrifuging at 2000 g for 10 minutes at 4°C. The plasma was aliquoted and stored at -70°C till tested. The cell pellet was preserved at -20°C for DNA extraction.

Screening coagulation tests (PT, APTT, TT) and fibrinogen assays were performed in a semi-automated coagulation analyzer (ST ART, Diagnostica Stago, Asniers, France). Plasma AT III was measured in a fully automated STA coagulation analyzer (STA Compact, Diagnositica Stago, France). ACLA (IgG, IgM), β2 GP1 (IgG/IgM), Annexin V (IgG/IgM) antibodies were measured by ELISA using commercial kits (Varelisa, Freiburg. Pharmacia). Protein C, total protein S were also measured by ELISA [Diagnostica Stago, Asniers, France]. The presence of lupus anticoagulant was assessed according to the criteria of International Society of Thrombosis & Hemostasis [8] by using screening and confirmatory reagents LA1 & LA2 (Dade Behring, Marburg Germany), Kaolin clotting time and dilute PT tests.

DNA was extracted as described earlier [9] from the cell pellet. Factor V Leiden, PT gene, MTHFR, Fibrinogen gene, EPCR and PAI 4G/5G polymorphisms were studied as described in earlier reports [10-15] with or without specific restriction enzyme digestion.

The assays for all the APA were repeated at least once with a minimum time interval of 3 months between the two tests while the PC, PS and AT III tests were also repeated again at least 3 months after the delivery and only the post delivery results were considered.

### Statistical analysis

Relative risks (RR), 95% CI and P values were estimated separately for each parameter and for the total number of samples using SPSS10 software.

## Results

Thirty two out of 34720 women presented with DVT during pregnancy giving a prevalence of 0.1% in this series. Seventeen presented with DVT in the first trimester, six in the second while 9 presented in the third trimester.

The sites of thrombosis among 32 women presented with DVT are shown in table 1.

**Table 1:** Sites of thrombosis in women with pregnancy associated DVT.

**Site of thrombosis**		**Number (%)**
**Lower limb DVT**	**with fetal loss**	09 (28.1)
	**without fetal loss**	13 (40.6)
	**Pregnancy induced hypertension (PIH) + DVT**	02 (6.25)
**Cortical venous thrombosis**		03 (9.3)
**Budd Chiary syndrome**		02 (6.25)
**Gangrene**		01 (3.1)
**Retinal vascular obstruction**		01 (3.1)
**Infra renal segment thrombosis**		01 (3.1)

The majority of the patients (75%) had lower limb DVT out of which 9 had history of fetal loss. Three women had cortical venous thrombosis while two were cases of Budd chiary syndrome. Retinal and renal vein thrombosis was observed in one case each and one was a case of gangrene.

The prevalence of acquired and genetic thrombophilia is shown in tables 2 &3.

**Table 2:** Prevalence of different antiphospholipid antibodies in pregnant women with DVT and normal pregnant controls

**APA**	**Patients (n = 32) Number positive (%)**	**Controls (n = 100) Number positive (%)**	**RR (95% CI)**	**P value**
**LA**	4 (12.5)	0	3.6 (1.6–4.5)	0.003
**ACLA**	10 (31.25)	1 (1)	4.5 (2.7–5.5)	0
**β2 GP 1**	2 (6.25)	1 (1)	2.1 (0.6–3.7)	0.22
**Annexin V**	5 (15.63)	1 (1)	3.8 (1.9–4.6)	0.003
**LA/ACLA/β2 GP 1/Annexin V**	12 (37.5)	3 (3)	7.4 (4.3–11.3)	0

**Table 3:** Genetic thrombophilia markers in patients and controls

**Thrombophilia marker**	**Patients (n = 32) Number positive (%)**	**Controls (n = 100) Number positive (%)**	**RR (95% CI)**	**P value**
**FVL Het**	4 (12.5)	0	4.0 (1.8–4.5)	0.001
**PT G20210A**	0	0	-	-
**EPCR Hets**	1 (3.13)	1 (1)	2.0 (0.3–3.8)	0.392
**MTHFR homo**	1 (3.13)	1 (1)	2.0 (0.3–3.8)	0.392
**MTHFR hetero**	6 (18.75)	12 (12)	1.46 (0.6–2.7)	0.333
**Fib β448 homo**	1(3.13)	1(1)	2.0 (0.3–3.8)	0.392
**Fib β448 hetero**	5 (15.63)	12 (12)	1.2 (0.5–2.5)	0.594
**PAI 4G/4G**	13 (40.63)	10 (10)	3.2 (1.8–5.1)	0
**4G/5G**	12 (37.5)	61 (61)	0.4 (0.2–0.8)	0.020
**5G/5G**	5 (15.63)	29 (29)	0.5 (0.2–1.1)	0.132
**PC Def**	1 (3.13)	0	2.8 (0.5–4.1)	0.223
**P S Def**	7 (21.88)	0	4.6 (2.6–5.6)	0
**AT III Def**	0	0	-	-

The IgG and IgM antibodies for ACLA and β2GPI were detected in 31.25% and 6.25% respectively while the prevalence of LA and anti annexin antibodies was found to be 12.5% and 15.63% respectively in the patients. In case of controls the prevalence of APA antibodies was 1% each for ACLA, β2GPI and annexin V while LA was not detected in any of the controls. Overall, 37.5% of the patients were found to be positive for any of the antiphospholipid antibodies/LA as against 3% among the normal pregnancy controls (RR 7.4 95% CI 4.3 – 11.3 P < 0.05).

Among the genetic thrombophilia markers, the heterozygous carrier rate for factor V Leiden was 12.5% as against 0% in controls (RR 4.57 95% CI 2.23–4.57 P < 0.05) while the frequency of EPCR and PAI 4G/4G homozygous state were 3.13% (RR 2.0; 95% CI 0.3–3.8 P NS) and 40.63 % (RR 3.2 ; 95% CI 1.8–5.1 P < 0.05), respectively. No difference in the prevalence was obtserved in case of MTHFR and fibrinogen gene polymorphisms between the controls and patients. Among the natural anticoagulants, protein S deficiency was a significant marker with 21.88% of the patients showing his deficiency (RR 4.6; 95% CI 2.6 – 5.6 P < 0.05) while Protein C deficiency was found to in one case (3.13%; RR 2.8 ; 95% CI 0.5–4.1 P NS). None of the cases and controls showed the presence of AT III deficiency and PT G20210A polymorphism.

Twenty two out of 32 (68.75%) patients showed the presence of two or more risk factors.

## Discussion

Pregnancy associated thrombosis is not a new condition as case reports of Phlegmasia Alba dolens (white leg of pregnancy) and Phlegmasia Coerulea dolens (Blue leg of pregnancy) can be found in text books of Obstetrics and Gynaecology since more than 70 years. However with introduction of more tests for thrombophilia markers and the ability to assess the variable relative risks of thrombosis associated with various thrombophilia markers along with better understanding of thrombohaemorrhagic balance in different thrombotic conditions, the subject is now being studied with renewed enthusiasm.

Maternal morbidity in India is quite high i.e around 4–5% [16,17]. Though the major cause of maternal morbidity in western countries is venous thromboembolism, in India haemorrhage, sepsis, complications during delivery, severe anaemia and toxaemia are the major causes of perinatal mortality [18,19]. Except one report on puerperial cerebral venous thrombosis [7], there is no data on the prevalence or the risk assessment for thrombosis during pregnancy from India.

Previous studies from western literature have reported incidence rates ranging from 18–95 events per 100000 women years during pregnancy [20-22]. The incidence in the present study is more or less the same. Moreover, the incidence of DVT in the present study is of symptomatic DVT and thus the prevalence obtained in the present study must be regarded as the minimum incidence rate. All these women have been followed up until delivery and those women who failed follow up have not been included in the study.

Presence of acquired thrombophilia i.e ACLA, β2 GPI and anti annexin V antibodies was observed in 37.5% of the women with a first objectivity confirmed episode of DVT in pregnancy with or without history of fetal loss. The relative risk for women with APA was much higher in pregnant women with DVT than that found in the normal control pregnant women.

Among the genetic thrombophilia, factor V Leiden mutation has been found to be the strongest risk factor as has been observed in several other studies [23-25]. The prevalence of factor V Leiden mutation in our general population and in deep vein thrombosis cases has been reported to be 2.3% and 2.6%, respectively [26]. In comparison, the mutation seems to be a strong risk factor in pregnant women presenting with DVT for the first time. The PT gene G20210A polymorphism has not been found in our control population or the disease groups thus is either extremely rare or nonexistent. The MTHFR C677T, fibrinogen β448 gene polymorphisms were not significantly different between the patients and the controls. The fibrinolysis gene polymorphism i,e PAI 4G/5G insertion/deletion – 675 bp upstream from the transcription start site has been found to play a key role with regulation of fibrinolysis and a few studies have shown an increased prevalence of 4G/4G polymorphism in cases of RSA [27]. Even in this study, 4G/4G homozygosity has been found to be a strong risk factor for DVT in pregnancy. Among the blood coagulation inhibitors i.e. PC, PS & ATIII deficiency, PS deficiency has been found to be strongly associated with DVT in pregnancy. PS deficiency has also been strongly linked to late pregnancy loss in several studies [28]. Another important information from this study is the presence of two or more factors causing thrombophilia in 22 out of 32 patients studied. The effect of pregnancy on some of the plasma factors like PC, PS and AT III has been ruled out by repeating these assays again at least 3 months after delivery.

In more than half of the women, DVT events occurred in the first trimester of the pregnancy. Higher frequency of DVT events has been reported during the first 15 weeks gestation [28]. Higher frequency of thromboembolic events in the first trimester of pregnancy provides an additional clue that when thromboprophylactic therapy has to be initiated it should begin much earlier in the pregnancy.

In pregnant women the common site of thrombosis was left leg. In a meta analysis published earlier it has been reported that about 82% of the objectively diagnosed DVT occurred in the left lower extremity. Though the exact cause is not known, anatomic reasons have been postulated [29,30].

What are the clinical implications of such a study? The important question always raised is whether all women with thrombophilia needs to be anticoagulated during pregnany. Anticoagulation therapy is certainly effective in reducing the incidence of adverse pregnancy outcomes in women with thrombophilia and one episode of DVT or history of idiopathic fetal loss. But is it justified to anticoagulate women with thrombophilia without any such adverse events as there are quite a few women who are carriers of thrombophilia and have successful pregnancy outcomes. Whether there should be an evaluation case by case or whether tailored therapy should be advised for specific thrombophilia need to be investigated. We still do not understand in quantitative terms why different thrombophilia markers have different impact in producing thrombosis. All these will remain as queries only until there are large studies on different antithrombotic modalities in prospective clinical trials.

## Competing interests

The author(s) declare that they have no competing interests.

## Authors' contributions

SV did the laboratory work. KG and SD contributed to the study design and manuscript preparation, VS and PS examined the patients. All authors read and approved the final manuscript.
